# Alternating expression levels of *WWOX* tumor suppressor and cancer-related genes in patients with bladder cancer

**DOI:** 10.3892/ol.2014.2476

**Published:** 2014-08-22

**Authors:** ELŻBIETA PŁUCIENNIK, MAGDALENA NOWAKOWSKA, ANNA STĘPIEN, MATEUSZ WOŁKOWICZ, ADAM STAWIŃSKI, WALDEMAR RÓŻAŃSKI, MAREK LIPIŃSKI, ANDRZEJ K BEDNAREK

**Affiliations:** 1 Department of Molecular Cancerogenesis, Medical University of Lodz, Lodz 90-752, Poland; 2 Laboratory of Clinical and Transplant Immunology and Genetics, Copernicus Memorial Hospital in Lodz, Lodz 93-513, Poland; 3 Bio-Tech Consulting Ltd., Lodz 90-212, Poland; 4 Second Department of Urology, Medical University of Lodz, Copernicus Memorial Hospital in Lodz, Lodz 93-513, Poland

**Keywords:** *WWOX* tumor suppressor, bladder cancer, reverse transcription-quantitative polymerase chain reaction, methylation, loss of heterozygosity

## Abstract

The aim of the present study was to determine the roles of the *WWOX* tumor suppressor and cancer-related genes in bladder tumor carcinogenesis. Reverse transcription-quantitative polymerase chain reaction was used to analyze the status of *WWOX* promoter methylation (using MethylScreen™ technology) and loss of heterozygosity (LOH) in papillary urothelial cancer tissues. The associations between the expression levels of the following tumorigenesis-related genes were also assessed: The *WWOX* tumor suppressor gene*,* the *MKI67* proliferation gene, the *BAX, BCL2* and *BIRC5* apoptotic genes, the *EGFR* signal transduction gene, the *VEGF* vascular endothelial growth factor gene, and the *CCND1* and *CCNE1* cell cycle genes. The results reveal a high frequency of LOH in intron 1 in the *WWOX* gene, as well as an association between reduced *WWOX* expression levels and increased promoter methylation. In addition, the present study demonstrates that in bladder tumors, apoptosis is inhibited by increased expression levels of the *BCL2* gene. A correlation between the proliferation indices of the *MKI67* and the *BIRC5* genes was also revealed*.* Furthermore, the expression levels of *VEGF* were identified to be positively associated with those of the *EGFR* gene.

## Introduction

Bladder cancer is the most common tumor of the urinary system. In 2010, bladder cancer was the third and thirteenth most commonly diagnosed type of cancer in males and females, respectively, in Poland (1).

Various carcinogenesis pathways in bladder cells have been proposed, including one which assumes that a single distinct molecular pathway exists for low-grade non-invasive tumors and another exists for muscle-invasive tumors. The first type of tumor develops from hyperplasia, and is characterized by molecular alternations in the RAF/MEK/ERK and PIK3CA signal transduction pathways, while the second, the muscle-invasive tumor type, progresses from a dysplastic urothelium that is characterized by disruptions in the *RB* and *p53* signaling pathways (2). Furthermore, chromosomal aberrations at a number of sites, including 1q, 5p, 17p, 3p, 13q, 18q and 10q are involved during carcinogenesis in bladder tissue. Epigenetic regulation of gene expression is also common and predominantly affects genes associated with tumor development and survival, such as *RUNX3, RASSF1A, p16, RARβ* and *CDH1* (2–5).

Genetic mapping and DNA sequencing has revealed the role of loss of heterozygosity (LOH) on chromosome 16 in the development of bladder cancer. Yoon *et al* (6) reported allelic loss at 16q24 in 20–45% of bladder tumors. This region overlaps with the fragile chromosomal site, FRA16D where the tumor suppressor gene, *WWOX* is located. Alterations in *WWOX* expression have been reported in various types of cancer, including breast (7), prostate (8), ovarian (9) and bladder cancer (10–12). However, the mechanisms responsible for the loss of *WWOX* expression remain unclear. The *WWOX* gene is not considered to be a classical tumor suppressor; for example, the two-hit model of cancer development, proposed by Knudson in 1985 (13), is not applicable. The susceptibility of *WWOX* to LOH, due to a location in a common fragile site, indicates that haploinsufficiency is a primary reason for reduced *WWOX* expression levels (14,15). Furthermore, epigenetic mechanisms have been proposed to be crucial in the regulation of *WWOX* expression (14).

The WWOX (WW-domain containing oxidoreductase) protein contains two N-terminal WW domains of protein-protein interactions and a C-terminal short-chain dehydrogenase domain (7). Numerous *WWOX* protein partners have been identified among the critical members of signal transduction pathways; proteins such as ERBB4 (16,17), JUN (18), TP73 (19), RUNX (20) and EZR (21).

In healthy tissues, high *WWOX* expression levels have been observed in endocrine organs, including the prostate, testis and mammary glands, which indicates involvement in sex hormone metabolism and the regulation of steroid signaling pathways (22). Furthermore, Aqeilan *et al* (23) observed that the *WWOX* gene is involved in the regulation of steroidogenesis and proper functioning of the gonads, i.e. the testis and ovary. *WWOX* knock-out mice exhibit downregulated expression of genes coding for enzymes in the cytochrome P450 family, including renin 1 structural and carbonyl reductase 2.

The aim of the present study was to analyze the alterations in mRNA expression levels of selected genes associated with proliferation (*MKI67*), apoptosis (*BCL2*, *BAX* and *BIRC5*), the cell cycle (*CCND1* and *CCNE1*), signal transduction (*EGFR* and *VEGF*) and tumor suppression (*WWOX)* in bladder tumor samples, and to identify any association between gene expression levels and clinicopathological factors, such as gender, grade or stage. The roles of promoter methylation status and LOH in the regulation of *WWOX* expression were also investigated.

## Materials and methods

### Tissue samples

Papillary urothelial cancer tissues were obtained from 32 patients treated at the Kopernik Hospital (Lodz, Poland) between 2003 and 2007. All patients had undergone transurethral resection of bladder tumors. The tumor tissue samples were stored at −80°C in RNAlater buffer (Ambion^®^; Thermo Fisher Scientific, Waltham, MA, USA).

The experimental group consisted of 26 males and six females. The tumors were graded according to the World Health Organization classification of Tumors (2004) (24) and staged using the tumor, node, metastasis (TNM) classification system. In the sample population, 16 tumors were classified as grade 1, nine as grade 2, three as grade 3 and four were unclassified. According to the TNM classification, 19 cases were non-invasive papillary carcinoma, five were T1, two were T2, one was T3 and five were unclassified. This study was conducted according to the Declaration of Helsinki and was approved by the Ethics Committee of the Medical University of Lodz (RMM/115/12/KE). Consent was obtained from the families of the patients.

### RNA and DNA isolation, and cDNA synthesis

RNA was isolated from the frozen tissue samples using TRIzol^®^ reagent (Invitrogen Life Technologies, Carlsbad, CA, USA). Reverse transcriptase from the ImProm Reverse Transcription (RT)-II™ system (Promega Corporation, Madison, WI, USA) was used to transcribe 10 μg total RNA to cDNA to obtain a final volume of 100 μl. The RT reaction was performed under the following conditions: Primer annealing at 25°C for 5 min, and elongation at 42°C for 60 min, followed by a 15 min pause in the reaction at 70°C. Following synthesis, 50 μl deionized water was added to each sample, which were stored at −20°C. Subsequent to RNA isolation, DNA was recovered using 0.5 ml back extraction buffer containing 1 M Tris Base, 4 M guanidinium thiocyanate and 50 mM sodium citrate, according to the manufacturer’s instructions.

### RT-quantitative polymerase chain reaction (qPCR)

Gene expression levels were analyzed using Rotor-Gene™ 6000 (Corbett Research, Cambridge, UK). The reaction products were detected using SYBR^®^ Green I and a qPCR Core kit for SYBR^®^ Green I (Eurogentec, Southampton, UK). Each reaction was performed in duplicate. The expression levels of the following genes were analyzed: *WWOX*, *MKI67*, *BAX*, *BCL2*, *BIRC5*, *EGFR*, *VEGF*, *CCND1,* and *CCNE1* and the results were compared with the expression levels of the *RPS17, H3F3A* and *RPLP0* reference genes. The primer sequences, PCR reaction conditions and the length of the obtained products are listed in Table I. Due to the low levels of the *WWOX* gene present in the tissue samples, semi-nested RT-qPCR was performed.

The primer sequences and the PCR conditions have been described in previous studies (25,26). Briefly, PCR cycling included one cycle at 95°C for 10 min (denaturation) followed by 35 cycles at 94°C for 30 sec (repeated denaturation); 56°C (for D16S3096) or 55°C (for D16S518) for 30 sec (annealing), and 72°C for 60 sec (elongation). In order to avoid detection of non-specific products for each reaction, melting curve analysis was performed and the expression levels of the genes were calculated according to the Roche method (27). Universal Human Reference RNA (Stratagene, La Jolla, CA, USA) at a concentration of 0.5 mg/ml served as a calibrator.

### LOH analysis

Allelic losses were analyzed by high resolution melting using a LightCycler^®^ 480 (Roche Diagnostics GmbH, Mannheim, Germany). Two microsatellite markers, located on chromosome 16 in two intron regions of the *WWOX* gene, were used: D16S3096 and D16S518 on introns 8 and 1, respectively. Information regarding the sequences for these microsatellite markers was obtained from the Genome Database (www.ncbi.nlm.nih.gov/probe?term=45798[unists+id]; www.ncbi.nlm.nih.gov/probe/?term=d16s518).

PCR cycling included one cycle at 95°C for 10 min, followed by 35 cycles at 94°C for 30 sec, 56°C (for D16S3096) or 55°C (for D16S518) for 30 sec, and 72°C for 60 sec.

### Methylation analysis of the *WWOX* gene

The methylation status of two fragments of the *WWOX* gene was analyzed; the first site in the promoter region between −508 and −174 bp, and the second between −171 and +239 bp, covering the 3′ end of the promoter and part of exon 1. The procedures for genomic DNA extraction, digestion and performing a MethylScreen™ assay (New England Biolabs, Hitchin, UK) have previously been described (25,26).

### Statistical analysis

A nonparametric Spearman linear correlation test was used in the analysis of the correlation between gene expression levels. The analysis of the dependence between *WWOX* gene expression levels and LOH, as well as methylation status and various clinical factors, was performed using the Aspin-Welsh test. P<0.05 was considered to indicate a statistically significant difference.

## Results

### LOH analysis

D16S518 and D16S3096 LOH was observed in 64.5 and 25.8% bladder cancer samples, respectively. Assuming that the population homozygosity values are 17% for D16S518 and 26% for D16S3096, according to the Genome Database, the predicted LOH were 47.5 and 0%, respectively. No correlation was observed between the LOH for either microsatellite loci, D16S518 or D16S3096, and the expression levels of the *WWOX* gene (P>0.05).

### WWOX methylation status

MethylScreen™ analysis revealed *WWOX* methylation in the −508 to −174 bp promoter region in 31% of bladder cancer specimens. Furthermore, *WWOX* expression levels in the methylated samples were almost half those of the unmethylated samples (means ± standard error of the mean [SE], 0.47±0.04 methylated vs. 0.90±0.14 unmethylated; P<0.05).

### Correlation between LOH and WWOX methylation status

**Figure 1:**
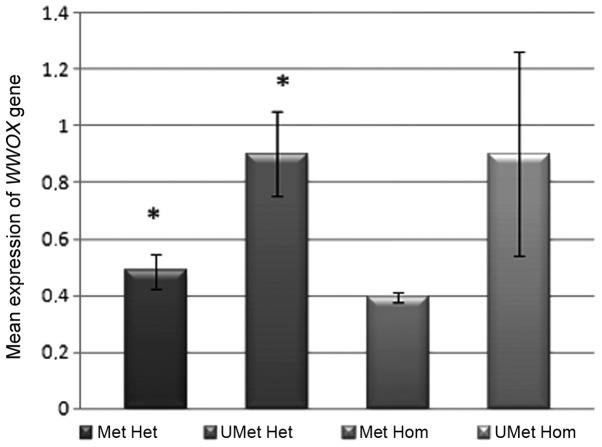
Mean expression levels of the *WWOX* gene associated with loss of heterozygosity in D16S3096 (marker located in intron 8 of the *WWOX* gene) and the methylation status of the promoter region between −508 and −174 bp (Aspin-Welsch test). ^*^P<0.05 Met Het vs. UMet Het. Met, methylated; UMet, unmethylated; Het, heterozygous; Hom, homozygous.

Promoter methylation in the −508 to −174 bp fragment appeared to reduce the expression levels of the *WWOX* gene in hetero- and homozygous cases of D16S3096 (Fig. 1). However, a statistically significant difference in the *WWOX* gene expression levels was observed between the heterozygous, unmethylated and the heterozygous, methylated samples (means ± SE, 0.90±0.15 vs. 0.49±0.06 respectively; P=0.019) at the D16S3096 locus, however, not at the D16S518 locus.

**Figure 2:**
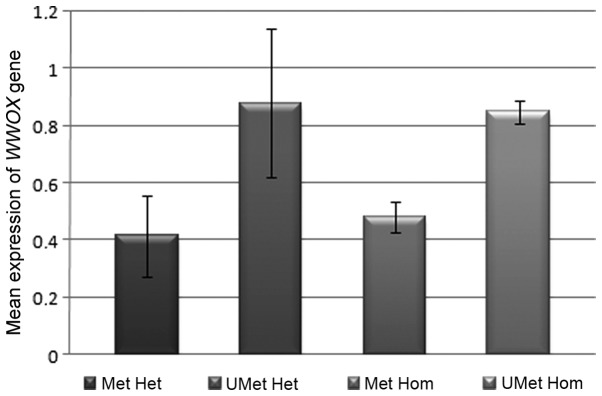
Mean expression levels of the *WWOX* gene associated with loss of heterozygosity in D16S518 (marker located in intron 1 of the WWOX gene) and the methylation status of the promoter region between −508 and −174 bp (Aspin-Welsch test). Met, methylated; UMet, unmethylated; Het, heterozygous; Hom, homozygous.

Similar associations between methylation and LOH in *WWOX* gene expression were observed for marker D16S518, located in intron 1 of the *WWOX* gene, although the results were not identified to be statistically significant; 0.88±0.26 for heterozygous, unmethylated vs. 0.41±0.14 for heterozygous, methylated (P=0.16), and 0.85±0.04 for homozygous, unmethylated vs. 0.48±0.05 for homozygous, methylated (P=0.07; Fig. 2).

### Correlation between genes

Numerous statistically significant correlations were identified between the selected genes associated with proliferation, apoptosis, cell cycle regulation and signal transduction. Significant positive correlations were observed between the expression levels of *BIRC5* (survivin) and those of the *MKI67* (R_s_=0.8170; P<0.0001) and *CCNE1* (R_s_=0.6578; P<0.0001) genes. Significant positive correlations were also observed between the expression levels of the *BIRC5* gene and two genes associated with signal transduction, *EGFR* and *VEGF* (R_s_=0.4753; P=0.006 and R_s_=0.3568; P=0.045, respectively). A positive correlation was also observed between the expression levels of the *MKI67* gene and the expression levels of *EGFR* (R_s_=0.3636; P=0.0408) and *CCNE1* (R_s_=0.7117; P<0.0001). Furthermore, a positive correlation was identified between the *EGFR* and *VEGF* expression levels (R_s_=0.5385; P=0.0015). The correlations between the expression levels of the analyzed genes are presented in Table II.

No statistically significant correlations between the expression levels of the above-mentioned genes and clinical factors, such as grade or stage were detected (P>0.05).

## Discussion

The molecular profile of bladder cancer has not been well recognized or described. However, numerous aspects of the underlying molecular mechanisms that affect the control of gene expression have been identified. LOH and methylation are processes that interfere with proper gene function. These mechanisms have been shown to be important in the regulation of the expression of *WWOX*, a tumor suppressor gene whose expression is frequently altered in a number of tumor types. This suppressor gene spans >1,000,000 base pairs and is located at 16q. The chromosomal section is characterized by frequent allelic losses in at least three regions. High frequencies of these deletions were initially observed in breast cancer samples (28). Further studies have revealed that LOH is associated with a reduction in *WWOX* expression in gastric (29), esophageal (15), pancreatic (14), lung (30) and breast cancer (31), as well as in glioblastoma multiforme (25). Examination of bladder cancer samples in the present study revealed that LOH occurred at a higher frequency in intron 1 (marker D16S518) than in intron 8 (marker D16S3096) of the *WWOX* gene: 64.5 vs. 25.8%, respectively. However, the influence of LOH on *WWOX* expression was considered to be negligible. Comparable results have been obtained for breast cancer (7). A similar percentage of *WWOX* gene LOH was also observed in pancreatic primary tumors (27%) (14), gastric carcinoma (31%) (29) and primary non-small cell lung cancer samples (37%) (30).

The present study also addressed the regulation of *WWO*X gene expression by an epigenetic mechanism, promoter methylation. The −508 to −174 bp region of the *WWOX* gene promoter was methylated in 31% of bladder cancer samples and was also associated with reduced levels of *WWOX* gene mRNA, which is consistent with results observed in glioblastoma multiforme specimens (25). Furthermore, regarding the D16S3096 locus, the present study revealed that the expression levels of the *WWOX* gene in unmethylated heterozygotes were almost twice of those in methylated heterozygotes; which was identified to be a statistically significant difference. Thus, methylation appeared to be the critical mechanism in the regulation of *WWOX* expression. Similar trends with regard to promoter methylation status were observed on intron 1, although no statistically significant differences between methylated and unmethylated hetero- or homozygotes were identified (D16S518; P>0.05).

A similar proportion of *WWOX* methylation has been observed in transitional carcinomas of the bladder (12). This hypermethylation within the *WWOX* promoter and exon 1 regions may be a result of cigarette smoking, a habit commonly observed in cancer bladder patients (10).

No statistically significant correlations between *WWOX* gene expression levels and clinical factors, such as grade and stage, were observed in the present study. However, an analysis of 101 primary bladder tumor samples by Ramos *et al* (11) revealed that lower levels of *WWOX* protein were significantly associated with a higher histological grade, as well as a more advanced stage, greater tumor size and further cancer progression. The authors proposed that *WWOX* may be a potential predictive marker for a more aggressive disease stage. However, a large number of tumor samples is required to demonstrate the influence of *WWOX* gene alternations on the clinical status of bladder cancer.

The present study used the molecular expression level profiles of bladder cancer samples to investigate the mRNA levels of cancer-related genes. Changes in the following cell cycle genes: Cyclin E1, the master regulator of progression from the the G_1_ to the S phase, and cyclin D1, which is associated with prompting entry into the cell cycle, were also evaluated. Shariat *et al* (32) demonstrated that a reduction in cyclin E1 expression levels was associated with an advanced tumor stage, lymph vessel invasion and lymph node metastases. In addition, reduced cyclin D1 and E1 expression levels were commonly correlated with altered expression levels of *pRB* and *p27*. The observed correlations were found to be associated with a poorer prognosis and reduced survival times in patients with bladder cancer. The results of the present study demonstrate a positive correlation between the levels of *CCND1* expression, and inhibition of apoptosis by the antiapoptotic *BCL2* gene and the *WWOX* tumor suppressor gene. Furthermore, a positive correlation was observed between the levels of *CCNE1* gene expression and the levels of inhibitor of apoptosis, *BIRC5,* accompanied by increased expression levels of the proliferation marker, *MKI67.* However, no association was identified between the levels of cyclin E1/D1 expression and clinical and pathological factors, such as grade or stage.

An imbalance in apoptosis may result in carcinogenesis in bladder tissues. However, the association between *BCL2* gene expression and cancer progression remains unclear. Shiina *et al* (33) demonstrate that increased expression levels of the *BCL2* gene are observed in numerous types of urinary tract cancers, are connected with a less aggressive tumor phenotype and are not significant in tumor progression. Conversely, Bilim *et al* (34) reported that high *BCL2* expression levels were associated with greater cancer progression. However, the results of the present study reveal a positive correlation between the expression levels of *BCL2* and *BAX* genes in bladder cancer.

A key process in bladder cancer progression is considered to involve a member of the inhibitor of apoptosis family, BIRC5 (survivin). Studies have demonstrated associations between overexpression of survivin and higher tumor stage, lymph node invasion (35,36) and possible shorter survival times (37).

The results of the present study demonstrate a marked positive correlation between the expression levels of *BIRC5*, *CCNE1* and *EGFR*. Furthermore, a dependence was observed between the levels of *BIRC5* mRNA and those of the *MKI67* marker of proliferation, which may indicate that cell survival and proliferation are closely associated in bladder cancer. Certain studies also report an association between increased *MKI67* expression levels and higher grade and stage (38). The results of the present study do not reveal any association between the expression levels of the genes regulating apoptosis (*BCL2*, *BAX* and *BIRC5*) and the *MKI67* gene that regulates proliferation, and clinical prognostic factors, such as grade or stage.

However, a marked positive correlation was observed between genes connected with angiogenesis (*VEGF*) and signal transduction (*EGFR*). Increased *VEGF* gene expression levels were observed in the transitional-epithelial type of bladder tumor and the expression levels were correlated with progression (39). These results are consistent with those of Crew *et al* (40), which demonstrated threefold higher V*EGF* expression levels in tumor samples, when compared with healthy bladder tissue samples. These results underline the value of *VEGF* in the prediction of disease recurrence and thus indicate an appropriate target for intravesical therapy. Previous studies have indicated that processes associated with the cell cycle, signal transduction, apoptosis and proliferation may be relevant in assessing the risk of local recurrence and survival rates in patients suffering from cancer of the bladder (41–46).

In addition, a significant increase in *EGFR* gene expression levels has been detected in invasive bladder tumors, compared with tumors of low malignancy, and *EGFR* has been associated with poor histological differentiation (47). However, in the present study, no association was observed between the expression levels of the above-mentioned genes, and gender, stage or grade.

The reduced expression levels of tumor suppressor genes may be one factor that initiates neoplastic lesion development. The results of the present study indicate that, in bladder cancer, *WWOX* gene expression levels may be reduced by two mechanisms: LOH or promoter region methylation. In this type of tumor, apoptosis may also be inhibited by increased expression levels of the *BCL2* gene. In addition, progression may be influenced as a result of the positive correlation between the *MKI67* proliferation index and the *BIRC5* apoptosis inhibitor gene expression levels.

In conclusion, the results of the present study provide an overview of the molecular changes that are apparent in bladder tumors. However, further molecular studies with a greater number of patients are required for an improved understanding of the biology of this disease and for the introduction of more effective therapeutic strategies.

## Figures and Tables

**Table I:** RT-PCR primer sequences.

Primer sequences (5′-3′)	Product length (bp)	Annealing temperature °(C)	Detection temperature (°C)
H3F3A	76	65	72
F: AGGACTTTAAAACAGATCTGCGCTTCCAGAG			
R: ACCAGATAGGCCTCACTTGCCTCCTGC			
RPLP0	69	65	72
F: ACGGATTACACCTTCCCACTTGCTGAAAAGGTC			
R: AGCCACAAAGGCAGATGGATCAGCCAAG			
RPS17	87	64	72
F: AAGCGCGTGTGCGAGGAGATCG			
R: TCGCTTCATCAGATGCGTGACATAACCTG			
MK167	156	56	81
F: TCCTTTGGTGGGCACCTAAGACCTG			
R: TGATGGTTGAGGCTGTTCCTTGATG			
BAX	137	56	81
F: AGAGGTCTTTTTCCGAGTGGCAGC			
R: TTCTGATCAGTTCCGGCACCTTG			
BCL2	122	56	81
F: TTGGCCCCCGTTGCTTTTCCTC			
R: TCCCACTCGTAGCCCCTCTGCGAC			
BIRC5	83	65	72
F: AGTGTTTCTTCTGCTTCAAGGAGCTGGAAG			
R: ACCGGACGAATGCTTTTTATGTTCCTCTATG			
EGFR	106	58	81
F: AGCTTCTTGCAGCGATACAGCTCAGAC			
R: TGGGAACGGACTGGTTTATGTATTCAGG			
VEGF	267	60	72
F: TGCTGTAGGAAGCTCATCTC			
R: ATCACGAAGTGGTGAAGTTC			
CCND1	160	03	86
F: TGTCCTACTACCGCCTCACACGCTTCCTCTCCAG			
R: TCCTCTTCCTCCTCCTCGGCGGCCTTG			
CCNE1	138	68	68
F: TTCTTGAGCAACACCCTCTTCTGCAGCC			
R: TCGCCATATACCGGTCAAAGAAATCTTGTGCC			
WWOX			
I step	171	63	72
F: TGCAACATCCTCTTCTCCAACGAGCTGCAC			
R: TCCCTGTTGCATGGACTTGGTGAAAGGC			
II step	150	63	77
F: GAGCTGCACCGTCGCCTCTCCCCAC			
R: TCCCTGTTGCATGGACTTGGTGAAAGGC			

F, forward; R, reverse.

**Table II:** Spearman rank correlation for selected genes in patients with bladder cancer.

Gene	Parameter	*CCNE1*	*EGFR*	*VEGF*	*MKI67*	*BCL-2*	*BAX*	*BIRC5*	*WWOX*
*CCND1*	R_s_ value	0.0689	0.3405	0.5830	0.2319	0.4661*	0.3430	0.0590	0.3780*
	P-value	>0.05	>0.05	>0.05	>0.05	0.0082*	>0.05	>0.05	0.0329*
*CCNE1*	R_s_ value		0.3101	0.2936	0.7117*	0.1435	0.2590	0.6578*	0.0002
	P-value		>0.05	>0.05	<0.0001*	>0.05	>0.05	<0.001*	>0.05
*EGFR*	R_s_ value			0.5385*	0.3636*	0.313	0.1960	0.4753*	0.1162
	P-value			0.0015*	0.0408*	>0.05	>0.05	0.0060*	>0.05
*VEGF*	R_s_ value				03047	0.1552	0.3598*	0.3568*	0.2038
	P-value				>0.05	>0.05	0.0431*	0.045*	>0.05
*MKI67*	R_s_ value					0.1076	0.0126	0.8170*	−0.0676
	P-value					>0.05	>0.05	<0.0001*	>0.05
*BCL-2*	R_s_ value						0.3849*	−0.0426	0.1424
	P-value						0.0296*	>0.05	>0.05
*BAX*	R_s_ value							0.0125	0.3018
	P-value							>0.05	>0.05
*BIRC5*	R_s_ value								−0.0896
	P-value								>0.05

* Indicates a statistically significant correlation.

R_s_, correlation coefficient.

## References

[1] Ministry of Health, Poland National Cancer Registry.

[2] Luis NM, López-Knowles E, Real FX (2007). Molecular biology of bladder cancer. Clin Transl Oncol.

[3] Kim WJ, Bae SC (2008). Molecular biomarkers in urothelial bladder cancer. Cancer Sci.

[4] Jung I, Messing E (2000). Molecular mechanisms and pathways in bladder cancer development and progression. Cancer Control.

[5] Mitra AP, Datar RH, Cote RJ (2006). Molecular pathways in invasive bladder cancer: new insights into mechanisms, progression, and target identification. J Clin Oncol.

[6] Yoon DS, Li L, Zhang RD, Kram A (2001). Genetic mapping and DNA sequence-based analysis of deleted regions on chromosome 16 involved in progression of bladder cancer from occult preneoplastic conditions to invasive disease. Oncogene.

[7] Bednarek AK, Laflin KJ, Daniel RL (2000). WWOX, a novel WW domain-containing protein mapping to human chromosome 16q23.3–24.1, a region frequently affected in breast cancer. Cancer Res.

[8] Qin HR, Iliopoulos D, Semba S (2006). A role for the WWOX gene in prostate cancer. Cancer Res.

[9] Lan C, Chenggang W, Yulan B (2012). Aberrant expression of WWOX protein in epithelial ovarian cancer: a clinicopathologic and immunohistochemical study. Int J Gynecol Pathol.

[10] Yang W, Cui S, Ma J (2012). Cigarette smoking extract causes hypermethylation and inactivation of WWOX gene in T-24 human bladder cancer cells. Neoplasma.

[11] Ramos D, Abba M, Lopez-Guerrero JA (2008). Low levels of WWOX protein immunoexpression correlate with tumour grade and a less favourable outcome in patients with urinary bladder tumours. Histopathology.

[12] Iliopoulos D, Guler G, Han SY (2005). Fragile genes as biomarkers: epigenetic control of WWOX and FHIT in lung, breast and bladder cancer. Oncogene.

[13] Knudson AG (1985). Hereditary cancer, oncogenes, and antioncogenes. Cancer Res.

[14] Kuroki T, Yendamuri S, Trapasso F (2004). The tumor suppressor gene WWOX at FRA16D is involved in pancreatic carcinogenesis. Clin Cancer Res.

[15] Kuroki T, Trapasso F, Shiraishi T (2002). Genetic alterations of the tumor suppressor gene WWOX in esophageal squamous cell carcinoma. Cancer Res.

[16] Aqeilan RI, Donati V, Palamarchuk A (2005). WW domain-containing proteins, WWOX and YAP, compete for interaction with ErbB-4 and modulate its transcriptional function. Cancer Res.

[17] Aqeilan RI, Donati V, Gaudio E (2007). Association of Wwox with ErbB4 in breast cancer. Cancer Res.

[18] Gaudio E, Palamarchuk A, Palumbo T (2006). Physical association with WWOX suppresses c-Jun transcriptional activity. Cancer Res.

[19] Aqeilan RI, Pekarsky Y, Herrero JJ (2004). Functional association between Wwox tumor suppressor protein and p73, a p53 homolog. Proc Natl Acad Sci USA.

[20] Aqeilan RI, Hassan MQ, de Bruin A (2008). The WWOX tumor suppressor is essential for postnatal survival and normal bone metabolism. J Biol Chem.

[21] Jin C, Ge L, Ding X (2006). PKA-mediated protein phosphorylation regulates ezrin-WWOX interaction. Biochem Biophys Res Commun.

[22] Nunez MI, Ludes-Meyers J, Aldaz CM (2006). WWOX protein expression in normal human tissues. J Mol Histol.

[23] Aqeilan RI, Hagan JP, de Bruin A (2009). Targeted ablation of the WW domain-containing oxidoreductase tumor suppressor leads to impaired steroidogenesis. Endocrinology.

[24] (2004). World Health Organization Classification of Tumours.

[25] Kosla K, Pluciennik E, Kurzyk A, Jesionek-Kupnicka D (2011). Molecular analysis of WWOX expression correlation with proliferation and apoptosis in glioblastoma multiforme. J Neurooncol.

[26] Płuciennik E, Nowakowska M, Wujcicka WI (2012). Genetic alterations of WWOX in Wilms’ tumor are involved in its carcinogenesis. Oncol Rep.

[27] Pfaffl MW, Horgan GW, Dempfle L (2002). Relative expression software tool (REST) for group-wise comparison and statistical analysis of relative expression results in real-time PCR. Nucleic Acids Res.

[28] Maeda N, Semba S, Nakayama S (2010). Loss of WW domain-containing oxidoreductase expression in the progression and development of gastric carcinoma: clinical and histopathologic correlations. Virchows Arch.

[29] Aqeilan RI, Kuroki T, Pekarsky Y (2004). Loss of WWOX expression in gastric carcinoma. Clin Cancer Res.

[30] Yendamuri S, Kuroki T, Trapasso F (2003). WW domain containing oxidoreductase gene expression is altered in non-small cell lung cancer. Cancer Res.

[31] Chen T, Sahin A, Aldaz CM (1996). Deletion map of chromosome 16q in ductal carcinoma in situ of the breast: refining a putative tumor suppressor gene region. Cancer Res.

[32] Shariat SF, Ashfaq R, Sagalowsky AI, Lotan Y (2006). Correlation of cyclin D1 and E1 expression with bladder cancer presence, invasion, progression, and metastasis. Hum Pathol.

[33] Shiina H, Igawa M, Urakami S (1996). Immunohistochemical analysis of Bcl-2 expression in transitional cell carcinoma of the bladder. J Clin Pathol.

[34] Bilim VN, Tomita Y, Kawasaki T (1998). Variable Bcl-2 phenotype in benign and malignant lesions of urothelium. Cancer Lett.

[35] Wang H, Xi X, Kong X (2004). The expression and significance of survivin mRNA in urinary bladder carcinomas. J Cancer Res Clin Oncol.

[36] Shariat SF, Ashfaq R, Karakiewicz PI (2007). Survivin expression is associated with bladder cancer presence, stage, progression, and mortality. Cancer.

[37] Kitamur H, Torigoe T, Honma I (2006). Expression and antigenicity of survivin, an inhibitor of apoptosis family member, in bladder cancer: Implications for specific immunotherapy. Urology.

[38] Gonzalez-Campora R, Davalos-Casanova G, Beato-Moreno A (2006). Apoptotic and proliferation indexes in primary superficial bladder tumors. Cancer Lett.

[39] Yang CC, Chu KC, Yeh WM (2004). The expression of vascular endothelial growth factor in transitional cell carcinoma of urinary bladder is correlated with cancer progression. Urol Oncol.

[40] Crew JP, O’Brien T, Bradburn M (1997). Vascular endothelial growth factor is a predictor of relapse and stage progression in superficial bladder cancer. Cancer Res.

[41] Pfister C, Moore L, Allard P, Larue H, Lacombe L, Têtu B, Meyer F, Fradet Y (1999). Predictive value of cell cycle markers p53, MDM2, p21, and Ki-67 in superficial bladder tumor recurrence. Clin Cancer Res.

[42] Behnsawy HM, Miyake H, Abdalla MA, Sayed MA, Ahmed Ael-F, Fujisawa M (2011). Expression of cell cycle-associated proteins in non-muscle-invasive bladder cancer: correlation with intravesical recurrence following transurethral resection. Urol Oncol.

[43] Chen L, Wang X, Mei H, Chen W (1998). Apoptosis and expression of PCNA in superficial transitional cell bladder cancer as related to recurrence. Zhonghua Wai Ke Za Zhi.

[44] Gazzaniga P, Gradilone A, Giuliani L, Gandini O, Silvestri I, Nofroni I, Saccani G, Frati L, Aglianò AM (2003). Expression and prognostic significance of LIVIN, SURVIVIN and other apoptosis-related genes in the progression of superficial bladder cancer. Ann Oncol.

[45] Jeong IG, Kim SH, Jeon HG, Kim BH, Moon KC, Lee SE, Lee E (2009). Prognostic value of apoptosis-related markers in urothelial cancer of the upper urinary tract. Hum Pathol.

[46] Chow NH, Liu HS, Lee EI, Chang CJ, Chan SH, Cheng HL, Tzai TS, Lin JS (1997). Significance of urinary epidermal growth factor and its receptor expression in human bladder cancer. Anticancer Res.

[47] Neal DE, Marsh C, Bennett MK (1985). Epidermal-growth-factor receptors in human bladder cancer: comparison of invasive and superficial tumours. Lancet.

